# Cheating at the Top: Trait Dominance Explains Dishonesty More Consistently Than Social Power

**DOI:** 10.1177/01461672211051481

**Published:** 2021-10-16

**Authors:** Kyoo-Hwa Kim, Ana Guinote

**Affiliations:** 1 University College London, UK; 2 Instituto Universitário de Lisboa (ISCTE-IUL), Portugal

**Keywords:** dominance, social power, dishonesty, power motivation, Covid-19

## Abstract

Power has long been associated with dishonesty. Here, we examined the contributions of personal and structural factors associated with power. Across five studies (*N* = 1,366), we tested the hypothesis that being dominant, more than having power and felt prestige, predicts dishonesty in incentivized tasks, moral disengagement, and breaking of Covid-19 containment rules. Dominance and dishonesty were positively associated (Study 1). Furthermore, dominance contributed to the positive relationship between occupational power and dishonesty in natural settings (Studies 2 and 5). Different types of power had inconsistent effects on dishonesty (Studies 3 and 4). Prestige was unrelated to dishonesty. Dominant individuals were overrepresented at the top, suggesting that the association between power and dishonesty may derive from self-selection processes, rather than power itself.

Dishonest power holders appear to be common. However, direct empirical evidence is nuanced (Lammers et al., 2015), and the links between power and dishonesty are not fully understood. It remains possible that misbehaviors of the powerful are simply more salient due to their oversized influence (Fiedler, 1991; Hamilton & Sherman, 1989). Here, we propose that the types of people who seek and attain power are disproportionally more dishonest. To shed light on factors implicated in dishonesty among powerful people, we separate the roles of predispositions that motivate people to seek and presumably attain power (trait dominance, feelings of prestige), and actual power’s effects on ethical conduct.

Dominance refers to the propensity to exhibit aggressive and fearless behavior in interpersonal relationships to pursue power and social advantages (Barrick et al., 2002; Maner & Case, 2016). It is a psychological trait that varies across individuals and is based on a system of biological, emotional, cognitive, and behavioral signatures—the Dominance Behavioral System (DBS; Johnson et al., 2012). The DBS facilitates the prioritization of attention and the deployment of any means necessary for people to ascend in social settings. This, we argue, includes dishonest means. Consequently, dominant people are overrepresented in authority positions. For instance, a study within the British civil service found that managers were more dominant compared with the general public, and this tendency increased as a function of manager seniority (Melamed & Bozionelos, 1992).

Dominant people often display assertive and forceful behavior (Mast et al., 2010). With a desire to outperform others and attain power (de Waal, 1986; Mehta et al., 2008), they can deceitfully signal competence (Anderson & Kilduff, 2009) and generate compliance from others (Cheng & Tracy, 2014), making them likely to achieve structural power. As such, trait dominance predicts the attainment of leadership positions (Judge et al., 2002). The promotion of dominant individuals is especially pronounced in competitive intergroup settings (Van Kleef et al., 2021), and under uncertainty, when individuals feel a lack of personal control (Kakkar & Sivanathan, 2017).

Dishonesty refers to behavior that violates prosocial norms (Gino & Mogilner, 2014) or socially accepted rules (Shu et al., 2011). Dishonesty is generally associated with high levels of motivations that accrue self-benefits, including performance motivation (Ames & Archer, 1988; Dweck, 1986) and feelings of entitlement (Stiles et al., 2018). Nevertheless, whether dominance triggers dishonesty remains unknown. This is an important gap, as dominance is overrepresented in power positions. We test the hypothesis that dominance is associated with dishonesty, even in contexts when seeking power is not at stake.

Dominance has been associated with innumerous self-serving inclinations, including narcissism (Bradlee & Emmons, 1992), hubristic pride, entitlement (Brown et al., 2009), and feeling fearless and invulnerable (Bronchain et al., 2019). Dominance is also associated with risky behavior (Demaree et al., 2009). Such inclinations could be proximal mechanisms that can justify and license dishonest behavior. If dominant people are disproportionately more deceitful than nondominant people and are more likely to attain power, their overrepresentation at the top could contribute to disproportionate observations of rule-breaking and unethical behavior among the powerful. A consideration of dominance as a predisposition that affords power, and of self-selection processes, is necessary for an understanding of the links between power and dishonesty frequently observed in ecological settings.

A second path to power emerges through status (Henrich & Gil-White, 2001; Maner, 2017). Status refers to attaining respect through prestigious attributes, such as competence or expertise (Durkee et al., 2020; Judge et al., 2004). It is often granted by others (Blader & Chen, 2014) and changeable (Hays & Bendersky, 2015). Prestige is frequently associated with various prosocial and selfless inclinations (Henrich et al., 2015; Ketterman & Maner, 2021), as well as authentic pride (Cheng et al., 2010). Dominance and prestige do not need to be based on actual power, but both assist social ascent (Cheng et al., 2013, 2021; Maner, 2017). Here to assess prestige, we relied on self-report of one’s felt prestige, which has been shown to be related to actual prestige (Cheng et al., 2010). We argue that feelings of prestige, unlike dominance, should not co-vary with dishonesty.

## Power, Dominance, and Dishonesty

With power comes the ability and authority to assert oneself over the social environment. Power enhances authenticity and self-expression (Guinote et al., 2002; Kraus et al., 2011), leading people to act more in line with their thoughts and inner states (Case & Maner, 2015; Pitesa & Thau, 2013). For instance, exchange oriented students (those who try to maintain their fair share of benefits) used power selfishly, but not communally oriented students (willing to benefit others) (Chen et al., 2001; Lee-Chai et al., 2001). Similarly, power amplifies an individual’s existing level of moral awareness (DeCelles et al., 2012).

Noteworthy is that past research investigating interactive effects between the person and situational power has primarily focused on predispositions that are independent of power (e.g., people’s relationship orientation). The joint influence of formal power (e.g., power roles in institutions) and predispositions that aid power attainment has been neglected in research. Dominant individuals exercise influence and control even in the absence of tangible power (Keltner et al., 2003). Consequently, their behavior could be less reliant on situational power. Moreover, dominance is a facet of extraversion (Hawley, 2002), linked to enhanced self-expression. Dominant individuals speak up and express their thoughts and feelings more readily. For instance, dominant individuals were more likely to interrupt others in interpersonal encounters, such as meetings, compared with nondominant individuals (Mast, 2002). A similar tendency occurs among those with social power (Anderson & Berdahl, 2002). Groups comprising powerful individuals have higher interpersonal variability, driven by their individuating self-expressions, compared with groups of individuals who lacked power (Guinote et al., 2002).

In summary, dishonesty among dominant individuals could be more frequent due to their reduced social inhibitions and self-serving inclinations that precede dishonesty. These individuals may license themselves to act unethically independently of holding power. Thus, the magnifying effects of power for self-expression (Guinote et al., 2012; Mead et al., 2018; Williams, 2014) may be less pronounced for dominance.

### Power and Dishonesty

Power is a situational ability to influence and control others, often (but not always) afforded by externally validated social structures (Carney, 2020). Power holders can influence others with meaningful and tangible means (French & Raven, 1959), such as rewards, punishments, or resources that others who are dependent on the power holder need (Fiske & Berdahl, 2007). Power emerges in relationships (Fiske & Dépret, 1996). As such, stable interpersonal processes, established via dominance and prestige, have been shown to play a crucial role in the acquisition of power (Cheng & Tracy, 2014).

Some studies point toward disproportionate dishonesty among the powerful. Leaders can demonstrate selfish behavior, such as taking from common resources (de Cremer & van Dijk, 2005). Powerful people can break rules (Van Kleef et al., 2011). Similarly, high socioeconomic status, a construct associated with power, predicts unethical behavior (Piff et al., 2012). Nevertheless, the links between power and dishonesty vary depending on the person and the context.

According to the situated focus theory of power (Guinote, 2007a, 2007b, 2008), predispositions and social context, such as power roles and organizational norms, influence power holders’ priorities. These factors activate goals that ultimately affect power holders’ ethical conduct, in a situated (Guinote & Chen, 2017; Guinote & Kim, 2020; Overbeck & Park, 2006) and nuanced (Fleischmann et al., 2019) manner. For instance, the construal of power may emphasize having power *over* others or *autonomy* (Lammers et al., 2009). Those who feel they have power over others are more likely to be aggressive and exploitative, unlike those with high perceived autonomy (Cislak et al., 2018). Similarly, when power is perceived as an opportunity, and not a responsibility (Sassenberg et al., 2014), power holders show more selfish behavior (Scholl et al., 2018). Context also influences power holders’ behavior. For example, a permissible organizational culture can foster or limit sexual harassment among the powerful (Pina et al., 2009).

## The Present Research

The present research first aims to set apart the roles of individual (dominance, prestige) and situational (tangible power) sources of power on ethical conduct. Given the connections between dominance and sentiments that predict dishonesty, we hypothesize that dominance will increase the use of dishonest strategies to advance one’s goals. Across studies, we operationalize dominance as forceful and assertive interpersonal behavior (Burgoon et al., 1998; Mast et al., 2010). Second, we test the notion that power holders frequently have dominant personalities. Thus, the association between power and dishonesty may derive from self-selection, rather than power itself. In contrast to dominance, prestige, another source of power, should not be associated with dishonesty. Finally, we explore the role of proximal self-beliefs that are closely aligned with dominance, such as entitlement and perceived invulnerability, which could allow dominant individuals to justify their dishonest behavior.

To assess dominance and prestige, we employed the dominance-prestige scale (Cheng et al., 2010), which has been validated and measures both prestige and dominance. Prestige is assessed through one’s own perception of enjoying respect and admiration, and dominance with coercive and assertive behavior. Study 1 established the relationship between dominance and dishonesty by giving participants the opportunity to cheat for personal gains. Studies 2 and 5 investigated the associations between dominance, occupational power, and dishonesty. To gather further data for the close association between dominance and power, Studies 3 and 4 focused on preferences for high or low power roles in a dyadic task, while manipulating power randomly. The studies gave participants the opportunity to morally disengage (Study 3) or to be dishonest (Study 4). To explore the possibility that the effects of power on ethical conduct are malleable and depend on the situation (Guinote, 2007a, 2008), power was assessed and manipulated in different contexts across studies.

Studies 1, 2, and 4 measured actual behavior during various tasks that gave participants the opportunity of acquiring monetary gains with dishonest means. Dishonesty was gauged incrementally (Studies 1, 3, 4, and 5) to mirror people’s tendency to cheat just a little, and not to the maximum (Mazar et al., 2008). Study 3 gauged moral disengagement as a proxy for dishonesty. To improve the generalizability of ethical decision-making, Study 5 measured unethical rule-breaking in participant’s actual daily lives during the Covid-19 pandemic. Ethics approval was obtained for all studies. Data files can be accessed at https://osf.io/v97dx/?view_only=2f16d0c0a308448f862a88fe0c01ca08.

## Study 1

This correlational study investigated the relationship between dominance and dishonesty. Dishonesty was inferred through an incentivized throw of a die (Fischbacher & Föllmi-Heusi, 2013; Hao & Houser, 2017). We hypothesized that the higher the dominance, the higher the die throw scores, implying a higher degree of dishonesty. In contrast, prestige should not be related to dishonesty.

### Method

#### Participants

In total, 211 students attending a European university participated. The sample size was predetermined, assuming (1 – β) = .90, α = .05, and ρ^2^ = .05 (.20 correlation). Seven participants were excluded for correctly guessing the study aims, leaving 204 participants (61 males, *M*_age_ = 20.12 years, *SD* = 2.18).

#### Procedure

Participants were informed that the study focused on social interactions. They completed the dominance-prestige scale (Cheng et al., 2010), and then they were given a die and asked to report the result of their throws (Haselhuhn & Wong, 2012). Participants provided feedback on their study experience, were checked for suspicion, debriefed, and given final opportunity to withdraw from the study.

#### Measures

##### Dominance and prestige

The dominance-prestige scale (Cheng et al., 2010) includes a subscale of eight items measuring dominance, such as “Others know it is better to let me have my way,” on 7-point Likert-type scales (1 = *strongly agree* to 7 = *strongly disagree*; α = .84). The prestige subscale includes eight items^1^ including “I am considered an expert on some matters by others” (α = .83).

##### Dishonesty

Participants were offered entry into a raffle with various prizes. They threw a die twice and were told that the sum of the two numbers they threw would correspond to the number of raffle tickets they would win. The experimenter and fellow participants were unable to see a participant’s throws to provide complete anonymity. While individual cheating remained unknown, dishonesty could be inferred from aggregate values, and a correlation between dominance level and reported die throw performance drawn.

### Results

Dominance was positively related to die scores *r*(204) = .255, *p* < .001. Male participants were higher in dominance than female participants, *M*_Male_ = 4.26, *SD*_Male_ = 1.232, *M*_Female_ = 3.64, *SD*_Female_ = 0.864, *t*(202) = 4.106, *p* < .001, *d* = 0.583. Dominance was positively correlated with prestige, *r*(204) = .223, *p* < .001, and older age, *r*(204) = .318, *p* < .001.

To assess the association between feelings of prestige and dishonesty and to rule out the influence of correlates of dominance, a multiple linear regression was carried out with dominance, prestige, their interaction, as well as age and gender as predictors and die performance as outcome variable. The overall regression was significant, *F*(5, 198) = 3.364, *p* = .006, *R*^2^ = .078, Cohen’s *f*^2^ = .085. Only dominance predicted dishonesty, *B* = .552, *p* = .004 (prestige: *B* = .192, *p* = .299; dominance × prestige: *B* = −.012, *p* = .934; gender: *B* = .248, *p* = .218; age: *B* = −.017, *p* = .840).

### Discussion

Dominance was related to dishonesty, while prestige was not. Study 1 found initial support for the notion that the more dominant participants are, the more likely they are to misreport higher scores to obtain prizes.

## Study 2

Study 2 investigated the relationship among dominance, prestige, occupational power, and dishonesty. The aims of the study were threefold. First, it tested the hypothesis that dominance predicts dishonesty, while felt prestige does not. Second, it established whether powerful roles are disproportionately occupied by dominant individuals. Finally and most importantly, it inspected whether the links between power and dishonesty are driven by higher dominance among power holders. Specifically, we hypothesized that both dominance and naturally occurring power would predict dishonesty. However, while dominance would predict dishonesty regardless of participants’ power, power would no longer predict dishonesty after controlling for dominance.

Study 2 was carried out through an online platform (prolific.co). Participants were employees in various industries, such as education or health care (Table S1), and reported their occupational power. Dishonesty was assessed via a virtual die throw.

### Method

#### Participants

In total, 194 adults in Europe participated. The sample size was predetermined, assuming (1 – β) = .90, α = .05, odds ratio = 2.8. Fifteen participants were excluded for suspicion, leaving a sample of 179 (*M*_age_ = 34.43 years, *SD* = 9.63). A majority identified themselves as females (*n* = 126) and Caucasian (*n* = 160).

#### Procedures

The study was introduced to participants as focusing on social interaction styles. Participants read that at the end of the survey a majority would proceed to an additional study, depending on a series of virtual die throws. Importantly, per minute, the alleged additional study was more lucrative (50% of base pay for 20% of time). This was used to provide a rationale and motivation for participants to cheat. Participants completed the dominance-prestige scale and then questions related to their hierarchical position at work (Kraus & Keltner, 2013).

#### Measures

##### Dominance and prestige

The dominance-prestige scale was used (8-item dominance α = .83, 9-item prestige α = .88).

##### Dishonesty

Participants virtually threw a die 5 times and reported the sum of values obtained. Participants were told they needed to throw 14 or more to qualify for the additional study, creating an incentive to inflate their result. In reality, the virtual die throw was preprogrammed to sum up to 12 (Dubois et al., 2015; Piff et al., 2012). As such, those who claimed to have thrown 14 or more were classified as having been dishonest.

##### Occupational power

We assessed two correlates of participants’ power position at their workplace: their relative position in an organigram (1 *= highest*, 7 = *lowest*) depicting the hierarchical structure of one’s organization, followed by a dichotomous question asking whether they had supervisory responsibilities at work (Supplemental Materials). Participants were classified as power holders if they fulfilled two conditions: have supervisory responsibilities and be in the middle or top level of the hierarchy (Levels 1–5 in the organigram). These criteria correspond to the common distribution of power in organizations across top and middle management (Raes et al., 2011).

### Results

The two measures of power were positively correlated ŋ = .441. Sixty-five participants (36.3%) were classified as powerful. Compared with those with low power (*n* = 114), powerful participants scored higher on dominance, *M*_HighPower_ = 3.50, *SD*_HighPower_ = 1.060, *M*_LowPower_ = 3.18, *SD*_LowPower_ = 1.010, *t*(177) = 1.988, *p =* .048, *d* = 0.310, and prestige, *M*_HighPower_ = 5.06, *SD*_HighPower_ = 0.833, *M*_LowPower_ = 4.75, *SD*_LowPower_ = 1.004, *t*(177) = 2.067, *p =* .040, *d* = 0.336. Dominance was positively correlated with prestige, *r*(179) = .159, *p* = .033.

Overall, 63 out of 179 participants (35.2%) were dishonest. A stepwise multiple binary logistic regression was used to test our hypotheses. Step 1 included power, and control variables age and gender. Step 2 added key predictors dominance and prestige. Step 3 included interaction variables between power, dominance, and prestige. In Step 1, power predicted dishonesty, *B* = .360, *Wald* = 4.751, *p* = .029, although the overall regression was not significant, χ^2^(3) = 5.688, *p* = .128. Adding dominance and prestige (Step 2) yielded an overall significant regression, χ^2^(5) = 14.774, *p* = .011. Dominance predicted dishonesty (*B* = .526, *Wald* = 8.483, *p* = .004), whereas power (*B* = .299, *Wald* = 3.019, *p* = .082) and prestige did not (*B* = −.030, *Wald* = 0.031, *p* = .861). In Step 3, the model fit worsened, χ^2^(9) = 16.285, *p* = .061, and only dominance (*B* = .519, *Wald* = 7.940, *p* = .005) predicted dishonesty (power × dominance: *B* = −.127, *p* = .469).

### Discussion

Powerful roles were disproportionately occupied by individuals who perceived themselves as dominant and prestigious. However, only dominance and not prestige predicted dishonesty. Consistent with hypotheses, while both dominance and naturalistic power predicted dishonesty, dominance predicted dishonesty above and beyond power. Power differences in dishonesty were no longer significant after controlling for dominance.

## Study 3

One strategy people use to distance themselves from their dishonest deeds is to morally disengage and interpret the unethical behavior as morally permissible (Bandura, 1996). The morally disengaged are more likely to commit unethical behavior (Barsky, 2008). Moral disengagement is therefore a proximal cognitive mechanism that facilitates dishonesty (Shu et al., 2011). As such, Study 3 investigated whether dominance, prestige, and power affect moral disengagement.

People in power positions often behave in an assertive, dominant manner. For instance, they talk and interrupt others, and readily take action (Galinsky et al., 2003; Guinote, 2017). It is therefore possible that elevated dominance observed among power holders in Study 2 was an effect of having power. To rule out this possibility and examine trait dominance independently of the experience of power, dominance and prestige were assessed 1 week prior to the study. Power was experimentally manipulated to examine its causal effects on dishonesty, when dominance and felt prestige are similarly distributed across power conditions. We hypothesized that dominance, but not prestige, or temporary experiences of power would predict dishonesty, measured by the tendency to morally disengage. Furthermore, power motivation was assessed. If dominant individuals are motivated to acquire power, they should prefer being in power compared with lacking power. A similar preference could exist for participants high on felt prestige.

### Method

#### Participants

In total, 146 university students based in Europe participated. The sample size was predetermined, assuming (1 – β) = .90, α = .05, and effect size *f*^2^ = .10. Five participants were excluded for correctly guessing the study aim, and we report findings from 141 participants (42 males; *M*_age_ = 21.49 years, *SD* = 3.45).

#### Procedures

Participants were told they would be participating in a study on decision-making and problem-solving in pairs. They completed measures of dominance, prestige, and demographics online. A week later, participants were assigned to dyads in the laboratory. To manipulate power, we adapted a procedure from Mast et al. (2010). Participants worked as a team on a simulated task. They were informed that one person would be the manager and the other would be the assistant. The experimenter remained blind to participants’ roles until later in the study. After managers chose a task for their assistants, participants discussed the task at a shared table.

Subsequently, participants went into individual cubicles to continue the study in private, where they stayed until they were dismissed. There participants completed the manipulation check and entitlement scale. Next, participants were given puzzles with an incentive to be dishonest, before they filled a questionnaire on moral disengagement. Participants provided feedback on their study experience and were checked for suspicion before receiving a detailed debrief.

#### Measures

##### Dominance and prestige

The dominance-prestige scale (Cheng et al., 2010) was completed (dominance α = .82, prestige α = .81), presented as a pre-questionnaire prior to the actual study.

##### Power manipulation

Participants were informed that the pre-questionnaire was a leadership questionnaire that determined their roles in the laboratory (Guinote, 2007b). In fact, participants were randomly assigned to their roles; half of the participants were assigned to be the art gallery manager (high power) and the remaining to be an assistant (low power). Role legitimacy was reinforced by informing participants about the experience and skills of managers, and the secondary nature of the assistant’s roles, thereby creating a difference in both power and status. Participants were informed that the manager would choose a task for their assistant. Although everyone would be entered into a lottery for vouchers, the assistants’ voucher amount would be determined by their managers’ evaluations. Therefore, managers controlled the outcomes of assistants and had tangible power (Fiske & Dépret, 1996).

##### Manipulation check

Participants indicated the degree to which they felt influential and in charge, on two-item, 7-point Likert-type scales (1 = *strongly disagree*, 7 = *strongly agree*).

##### Moral disengagement

First, dishonesty was potentially permitted. Participates were informed of the potential to complete the study considerably quicker. All participants were presented with six spatial puzzles, allegedly to measure their problem-solving capabilities. Unbeknownst to them, only three puzzles were solvable (Pulfrey & Butera, 2013). Participants read that if they solved four or more puzzles, they would be able to skip a second test (Flynn et al., 1987). According to a pretest (*n* = 38), students were strongly opposed to lying in university premises, and the rate of dishonesty was too low for it to be a sensitive measure. Therefore, rather than asking how many puzzles they solved, participants completed the moral disengagement questionnaire (six items, 7-point Likert-type scales, α = .70), right after being exposed to the temptation to cheat under the puzzle paradigm. An example item is, “It is appropriate to seek short-cuts as long as it is not at someone else’s expense.” This questionnaire has been validated in previous research (Moore et al., 2012; Shu et al., 2011).

##### Power motivation

Participants indicated their enjoyment and perceived suitability of their assigned role on two 7-point Likert-type scales.

### Results

#### Manipulation check

Seventy-two participants (51.1%) were assigned to the manager role. An independent-samples *t* test showed no significant differences in age, gender, race, or English proficiency between managers and assistants. Participants’ perceptions of their influence and control were collapsed into one measure, *r*(141) = .721, *p <* .001. The managers claimed to feel more powerful than the assistants, *M*_Manager_ = 5.76, *SD*_Manager_ = 1.058, *M*_Assistant_ = 4.27, *SD*_Assistant_ = 1.492, *t*(139) = 6.891, *p <* .001, *d* = 1.152. Hence, the power manipulation was deemed effective.

#### Power motivation

The measures of participant’s enjoyment and perceived suitability of their roles were collapsed into one score of role preference, *r*(141) = .797, *p <* .001. A multiple linear regression with power, dominance, prestige, and their interactions as predictors was overall significant, *F*(7, 133) = 6.787, *p* < .001, adjusted *R*^2^ = .224. Both power × dominance (*B* = .485, *p* < .001) and power × prestige (*B* = .233, *p* = .027) influenced role preferences. Dominance was positively related to prestige, *r*(141) = .194, *p =* .021.

For participants assigned to the manager role, higher levels of dominance (*B* = .387, *p* = .027, Table 1) and felt prestige (*B* = .555, *p* < .001; Table S2) coincided with higher perceived enjoyment and suitability of the role. For participants assigned to the assistant role, higher levels of dominance was associated with lower preference (*B* = −.605, *p* < .001) of their assigned role (prestige: *B* = .093, *p* = .566). Thus, participants enjoyed and preferred positions that were congruent with their dominance level.

**Table 1.** Role Preference by Power and Dominance—Studies 3 and 4.

Study 3				95% confidence interval	
Dominance level	Power condition	Role preference	*SE*	Lower bound	Upper bound
High	High	5.393	.230	4.938	5.848
High	Low	4.759	.216	4.331	5.188
Low	High	5.396	.212	4.976	5.816
Low	Low	5.847	.227	5.397	6.296
Study 4				95% confidence interval	
Dominance level	Power condition	Role preference	*SE*	Lower bound	Upper bound
High	High	5.323	.251	4.828	5.818
High	Low	4.750	.235	4.285	5.215
Low	High	4.341	.245	3.858	4.823
Low	Low	4.697	.214	4.275	5.118

*Note.* Role preference on 7-point Likert-type scale. Higher mean indicates higher preference for the power condition.

#### Moral disengagement

A stepwise multiple linear regression included power, age, and gender in Step 1. Step 2 added key predictors dominance and prestige. Step 3 included control variable role preference. Step 4 added interaction variables between power, dominance, and prestige. Step 1 was significant, *F*(3, 137) = 4.048, *p* = .009, adjusted *R*^2^ = .061, showing that the powerful displayed *lower* levels of moral disengagement, although this did not reach significance following conventional threshold levels (*B* = −.146, *p* = .052). Being male (*B* = .240, *p* = .004) was associated with higher moral disengagement. Adding dominance and prestige in Step 2 contributed to explaining the outcome variance, significant Δ*F* = .006, *F*(5, 135) = 4.735, *p* < .001, adjusted *R*^2^ = .118. Dominance predicted higher moral disengagement, *B* = .234, *p* = .005, whereas prestige was linked to *lower* moral disengagement, *B* = −.173, *p* = .036. The negative effect of power condition remained, *B* = −.167, *p* = .038, along with being male, *B* = .247, *p* = .006. Neither Step 3—significant Δ*F* = .818, *F*(6, 134) = 3.927, *p* < .001, adjusted *R*^2^ = .111—nor Step 4 improved the model (Table 2). Neither role preference (*B* = −.007, *p* = .915) nor power × dominance predicted moral disengagement (*B* = −.008, *p* = .928).^2^

**Table 2.** Stepwise Regression on Moral Disengagement—Study 3.

Model	*R*	*R* ^2^	Adjusted *R*^2^	*SE* of the estimates	Change statistics				
					Δ*R*^2^	Δ*F*	*df*1	*df*2	Sig. Δ*F*
1	.285	.081	.061	.9689	.081	4.048	3	137	.009
2	.386	.149	.118	.9393	.068	5.376	2	135	.006
3	.387	.150	.111	.9426	.000	0.053	1	134	.818
4	.393	.155	.090	.9541	.005	0.201	4	130	.938

*Note.* 1. Predictors: (constant), age, power, gender. 2. Predictors: (constant), age, power, gender, dominance, prestige. 3. Predictors: (constant), age, power, gender, dominance, prestige, role preference. 4. Predictors: (constant), age, power, gender, dominance, prestige, role preference, power × dominance, power × prestige, dominance × prestige, power × dominance × prestige.

### Discussion

Dominance was related to higher levels of moral disengagement. In contrast, power had the opposite effect, as managers tended to morally engage. Although not tested, it is possible that the elevated status of power holders elicited responsibility, and consequently honesty. Prestige was related to lower levels of moral disengagement. Despite being positively correlated with one another, dominance and prestige demonstrated opposite associations to moral disengagement. Both individuals high in prestige and dominance strive for power, but their attitudes in the moral domain diverged.

## Study 4

Study 4 tested the hypothesis that dominance, more than power or prestige, predisposes individuals to be dishonest for direct and immediate monetary gains. The study employed a different power manipulation from Study 3. Power was manipulated with a commonly used recall exercise (Galinsky et al., 2003), enabling the simulation of varied experiences of power, which are not necessarily linked to formal positions.

Dishonesty was assessed with the same puzzle task used in Study 3; however, here participants actually reported their performance. Unlike Study 3, Study 4 was conducted online, and participants did not share any association with the university. In addition, performance motivation was assessed and controlled for, in consideration of the nature of the outcome variable: performance in a puzzle, ostensibly a measure of skill rather than luck. Preferences for power were assessed. We hypothesized that dominance would predict a preference for power.

### Method

#### Participants

We recruited U.K. based working adults online (prolific.co). We collected data in two stages. In total, 220 adults participated in the first stage, and 180 participated in the second stage. The sample size was predetermined assuming (1 – β) = .90, α = .05, and effect size *f*^2^ = .10. Two participants were excluded for correctly guessing the study aim, leaving 178 participants (66 males; *M*_age_ = 35.58 years, *SD* = 11.16). Participants were randomly assigned to high or low power conditions. Eighty-one participants (45.5%) were assigned to the powerful condition. Participants received £2 and a bonus compensation.

#### Procedure

The study was introduced to participants as focusing on social interactions. In Stage 1, participants reported their chronic predispositions. Stage 2 took place 7 to 10 days later. Participants wrote about a past event, alleged as a memory recall exercise. They then completed a manipulation check, followed by a question on enjoyment. Participants proceeded to solve puzzles, which were ostensibly unrelated to the writing exercise (Pulfrey & Butera, 2013). Participants read they would receive a bonus depending on the number of puzzles solved. After reporting their performance, participants indicated how motivated they were.

#### Measures

##### Dominance and prestige

The dominance-prestige scale was used (dominance α = .80, prestige α = .85).

##### Power manipulation

Participants wrote a short essay (Galinsky et al., 2003). Half of the participants were asked to write about an experience when they had power over another person (high power), and the other half wrote about when another person had power over them (low power). Participants were asked to write in detail and as vividly as possible.

##### Manipulation check

The same two items, *r*(178) = .794, *p <* .001, from Study 3 were used.

##### Dishonesty

Participants were presented with six puzzles (Pulfrey & Butera, 2013), to receive a bonus depending on their claimed performance. Their bonus would increase by £0.10 per every puzzle, up to £0.50 for solving all puzzles. Because three puzzles were solvable, we deemed all participants who solved three or less as being honest, and re-coded their scores to 3. Thus, we were left with a continuous variable that measured cheating behavior with 3, 4, 5, and 6 as possible values.

#### Performance motivation

One question adapted from Van Yperen et al. (2011) measured participants’ performance motivation (Supplemental Materials).

##### Power motivation

Participants indicated how much they enjoyed the writing task on a 7-point Likert-type scale.

### Results

Across power conditions, participants did not significantly differ in gender, race, or age. There was no significant difference in the stated enjoyment of the task between power conditions, *M*_HighPower_ = 4.83, *SD*_HighPower_ = 1.611, *M*_LowPower_ = 4.71, *SD*_LowPower_ = 1.534, *t*(176) = 0.490, *p* = .625.

#### Manipulation check

Participants assigned to the high power condition felt more in control and influential compared with those assigned to the low power condition, *M*_HighPower_ = 6.00, *SD*_HighPower_ = 0.879, *M*_LowPower_ = 2.67, *SD*_LowPower_ = 1.373, *t*(176) = 18.837, *p <* .001, *d* = 2.889.

#### Power motivation

A multiple linear regression with power, dominance, prestige, and their interactions as predictors, and role enjoyment as the outcome variable, *F*(7, 170) = 1.383, *p* = .215, showed no significant power × dominance (*B* = .143, *p* = .238; Table 1) or power × prestige interaction (*B* = −.116, *p* = .331; Table S2). A closer inspection showed that for individuals assigned to the high power condition, higher dominance tended to be associated (*B* = .335, *p* = .058) with the tendency to enjoy the recall task (prestige: *B* = .092, *p* = .603). For those assigned to the low power condition, high prestige (*B* = .349, *p* = .030) was associated with enjoyment of the recall task (dominance: *B* = .050, *p* = .751).

#### Dishonesty

A stepwise multiple linear regression was used to test our hypotheses. Step 1 included power and control variables age and gender. We added key predictors dominance and prestige in Step 2. Step 3 included control variables enjoyment and performance motivation to assess whether dominance predicts dishonesty over and above these variables. Finally, we added interaction variables between power, dominance, and prestige (Step 4) to explore moderation effects. Step 1 did not reach significance, *F*(3, 174) = 1.008, *p* = .391, and power did not explain the variance in dishonesty, *B* = .068, *p* = .396. After adding dominance and prestige in Step 2 (significant Δ*F* = .055), the overall regression was still not significant, *F*(5, 172) = 1.799, *p* = .115. Nevertheless, dominance predicted dishonesty, *B* = .162, *p* = .046.

Step 3 was overall significant, *F*(7, 170) = 2.150, *p* = .041, adjusted *R*^2^ = .044, significant Δ*F* = .056. Dishonesty was related to performance motivation, *B* = .103, *p* = .035. Dominance did not reach conventional threshold levels for significance, *B* = .139, *p* = .088. No other variables approached significance (power: *B* = .054, *p* = .495; prestige: *B* = .054, *p* = .500; enjoyment: *B* = .044, *p* = .394; age: *B* = −.005, *p* = .532; male: *B* = −.055, *p* = .506). Step 4 did not contribute to the model (Table S4), and power × dominance (*B* = −.010, *p* = .907) was not significant.

Although dominance was related to dishonesty, this relationship became nonsignificant when controlling for performance motivation, which was positively related to dominance, *r*(178) = .164, *p* = .028, and dishonesty, *r*(178) = .207, *p* = .006. Performance motivation was unrelated to prestige, *r*(178) = .117, *p* = .121 (Table 3).^3^

**Table 3.** The Associations of Dishonesty and Dominance, Prestige, and Performance Motivation—Study 4.

		Dishonesty	Dominance	Performance motivation
Dominance	Pearson correlation	.180*	1	
	Sig.	.016		
	*N*	178	178	
Performance motivation	Pearson correlation	.207**	.164*	1
	Sig.	.006	.028	
	*N*	178	178	178
Prestige	Pearson correlation	.094	.106	.117
	Sig.	.214	.160	.121
	*N*	178	178	178

* Correlation is significant at the 0.05 level (2-tailed)

** Correlation is significant at the 0.01 level (2-tailed)

### Discussion

Dominance was positively associated with dishonesty. In contrast, power and prestige were unrelated to dishonesty. Dominance tended to be associated with enjoyment of recalling experiences of power. Consistent with past research, dominance was related to performance motivation, which predicted dishonesty.

## Study 5

Studies 1 to 4 investigated dishonesty or rule-breaking behavior that benefits the self in the form of money and time. The social consequences of cheating were trivial. To complement, Study 5 focused on common daily wrongdoings, with social consequences, such as harm to others. Specifically, it focused on breaking of lockdown rules imposed by the government to contain the spread of Covid-19.

First, Study 5 tested whether dominant individuals are more likely to break lockdown rules, an offense that endangers others. This tendency would not apply to individuals high in felt prestige. Second, as in Study 2, Study 5 tested the hypothesis that occupational power is disproportionately occupied by dominant individuals, and that the links between power and Covid-19 offenses should predominantly be driven by elevated dominance among power holders. Specifically, we hypothesized that the association between occupational power and rule-breaking behavior would not be significant after controlling for dominance. Finally, Study 5 sought to find exploratory information for cognitive representations that would license dominant individuals to break rules: entitlement and perceived invulnerability to Covid-19.

Study 5 was an online field survey. Participants were recruited through community groups within a large European city. Data were collected within a 3-week period in July and August 2020. Demographic information and other control variables that could influence rule-breaking, such as the prevalence of Covid-19 in a participant’s local area, were assessed.

### Method

#### Participants

In total, 678 adult members of local Facebook groups participated. Fourteen were excluded for suspicion, leaving 664 participants (*M*_age_ = 45.17 years, *SD* = 12.95). A majority of the respondents were females (*n* = 573, 86.3%), Caucasian (*n* = 565, 85.1%), and employed (*n* = 500, 75.3%).

#### Procedures

The study was introduced as focusing on decision-making during the Covid-19 pandemic. Participants completed a questionnaire that measured dominance, prestige, entitlement, and perceived vulnerability to Covid-19. One question checked whether participants were paying attention. Participants’ past behavior between March 23 and June 15, 2020, was assessed. During this period, the government had imposed strict rules to limit social contact. Then, we asked participants about their future behavior. Power was assessed through the presence or absence of supervision responsibilities. Finally, participants provided feedback on their experience and received a detailed debrief, before giving final consent.

#### Measures

##### Dominance and prestige

Participants completed the dominance-prestige scale (dominance α = .79, prestige α = .80; Cheng et al., 2010).

##### Rule-breaking

Participants answered six questions regarding their past behaviors. The questions covered unlawful behavior, such as the degree to which participants had left their home for *un*essential activities (5-point Likert-type scale, 1 = *never*, 5 = *more than 3 times*), or adhered to social distancing (reverse coded; 7-point Likert-type scale, 1 = *all the time*, 7 = *never*). All items were standardized (6-item α = .62), and subsequently a single score of past rule-breaking was constructed. Participants then reported their planned behavior for the next 4 weeks. The questions were adapted to reflect rule changes. Examples include the intention to wear face coverings (reverse coded) or attend large gatherings (7-point Likert-type scale, 1 = *extremely unlikely*, 7 = *extremely likely*). Answers were standardized (4-item α = .59) and collapsed into one score of planned rule-breaking.

##### Occupational power

Participants in work indicated whether they held a supervisory or leadership position at work (*yes*/*no*).

##### Control variables

Demographic information such as age, gender, race, education level, and household income was assessed. Participants reported whether they had preexisting medical conditions that would make them more likely to suffer from Covid-19. Participants rated on a 5-point Likert-type scale (1 = *much fewer*, 5 = *many more*) the level of local Covid-19 prevalence. In addition, we assessed proximal beliefs associated with dominance: entitlement and perceived vulnerability. Feelings of entitlement were assessed with the psychological entitlement scale (PES). The scale contained eight items, such as “I demand the best because I am worth it,” on 7-point Likert-type scales (Campbell et al., 2004; α = .82). Participants’ perceived vulnerability to contracting and suffering from Covid-19 was assessed with nine items, adapted from the perceived vulnerability to disease scale (Ahorsu et al., 2020; Duncan et al., 2009). An example is, “It is unlikely I will catch Coronavirus, even if it is going around (reverse coded).” Participants indicated their level of agreement (1 = *strongly disagree*, 7 = *strongly agree*; α = .76).

### Results

#### Rule-breaking

Those who had power at work (*n* = 293) were more dominant compared with those who did not have power at work (*n* = 207), *M*_HighPower_ = 3.30, *SD*_HighPower_ = 0.912, *M*_LowPower_ = 2.96, *SD*_LowPower_ = 0.835, *t*(498) = 4.297, *p <* .001, *d* = 0.393. They also scored higher on prestige, *M*_HighPower_ = 5.19, *SD*_HighPower_ = 0.667, *M*_LowPower_ = 5.00, *SD*_LowPower_ = 0.765, *t*(498) = 2.952, *p* = .003, *d* = 0.265. Crucially, occupational power was associated with higher levels of rule-breaking, *standardized M_H_*_ighPower_ = 0.0901, *SD*_HighPower_ = 0.585, *M*_LowPower_ = −0.0155, *SD*_LowPower_ = 0.525, *t*(498) = 2.073, *p* = .039, *d* = 0.190.

A stepwise multiple linear regression was employed. Step 1 included power and control variables age, gender, education, income, preexisting conditions, and local Covid-19 prevalence. We added dominance and prestige in Step 2. Step 3 added interaction variables between power, dominance, and prestige. Finally, in Step 4, feelings of entitlement and perceived vulnerability to Covid-19 were added as covariates. Step 1 was significant, *F*(7, 419) = 5.244, *p* < .001, adjusted *R*^2^ = .065, showing that power predicted higher levels of rule-breaking (*B* = .121, *p* = .035). Step 2 improved the model further, significant Δ*F* = .048, *F*(9, 417) = 4.799, *p* < .001, adjusted *R*^2^ = .074. Dominance predicted rule-breaking (*B* = .065, *p* = .018), whereas prestige did not (*B* = .013, *p* = .633), and the association between power and rule-breaking was no longer significant (*B* = .103, *p* = .073).

Step 3 did not improve the model, significant Δ*F* = .109, *F*(13, 413) = 3.937, *p* < .001, adjusted *R*^2^ = .082. Increased rule-breaking was associated with younger age (*B* = −.009, *p* < .001) and not having a preexisting medical condition (*B* = −.184, *p* = .004). The interaction power × dominance approached, but did not reach statistical significance (*B* = .110, *p* = .053). Specifically, among those with work power, dominance predicted breaking of Covid-19 containment rules, *F*(1, 291) = 13.108, *B* = .122, *p* < .001. No such effects were found among those without work power, *F*(1, 205) = 0.021, *p* = .886. In Step 3, neither power (*B* = .106, *p* = .064) nor dominance (*B* = −.005, *p* = .910) were uniquely related to rule-breaking.

Step 4 improved the model further, significant Δ*F* = .001, *F*(15, 411) = 9.0814, *p* < .001, adjusted *R*^2^ = .222 (Table 4). Work power (*B* = .097, *p* = .066), dominance (*B* = −.039, *p* = .365), and their interaction variable (*B* = .089, *p* = .093) were not significantly associated with rule-breaking. Feeling entitled (*B* = .070, *p* = .011) and *in*vulnerable to Covid-19 (*B* = −.209, *p* < .001) coincided with rule-breaking behavior.^4^

**Table 4.** Power, Dominance, and Prestige on Rule-Breaking—Study 5.

Model	*R*	*R* ^2^	Adjusted *R*^2^	*SE* of the estimate	Change statistics				
					Δ*R*^2^	Δ*F*	*df*1	*df*2	Sig. Δ*F*
1	.284	.081	.065	.54118	.081	5.244	7	419	.000
2	.306	.094	.074	.53854	.013	3.061	2	417	.048
3	.332	.110	.082	.53622	.016	1.903	4	413	.109
4	.499	.249	.222	.49386	.139	37.943	2	411	.000

*Note.* 1. Predictors: (constant), age, gender, power, borough, education, medical condition, income. 2. Predictors: (constant), age, gender, power, borough, education, medical condition, income, prestige, dominance. 3. Predictors: (constant), age, gender, power, borough, education, medical condition, income, dominance, prestige, dominance × power, dominance × prestige, power × prestige, dominance × power × prestige. 4. Predictors: (constant), age, gender, power, borough, education, medical condition, income, dominance, prestige, dominance × power, dominance × prestige, power × prestige, dominance × power × prestige, entitlement, invulnerability.

### Discussion

Study 5 found that dominant individuals are more likely to break Covid-19-related rules compared with nondominant individuals. They were also more entitled and felt less vulnerable to Covid-19. Prestige did not affect rule-breaking. Powerful roles were disproportionately occupied by dominant individuals and those high in felt prestige. Those with occupational power were more likely to offend, but crucially, power no longer predicted rule-breaking after controlling for dominance.

## General Discussion

Power has extensively been associated with corruption and dishonesty. Here, we provided a differentiated examination of this relationship, considering both predispositions that afford power in natural settings and tangible power. Our aim was to explore the self-selection processes that may trigger disproportionate dishonesty among power holders. We hypothesized that dominance, but not power or prestige, would be related to dishonesty. This would occur because dominant individuals strive to accrue self-serving benefits (Boehm & Flack, 2010), such as time, money, or freedom from constraints, with disregard for social rules (Shu et al., 2011). We further hypothesized that dominant individuals would strive for power (Barrick et al., 2002; Mast et al., 2010) and be overrepresented at the top (Lord et al., 1986), contributing to the links between power and dishonesty.

Across five studies, dominance was consistently associated with dishonesty (Table 5). The hypothesis that dominant individuals desire power, and are more likely to obtain power, was supported. Dominance was by and large associated with a conscious enjoyment of and desire for power (Studies 3 and 4). Furthermore, dominant individuals disproportionally resided in positions of power, which contributed significantly to the relationship between power and dishonesty (Studies 2 and 5). These findings demonstrate that dishonesty is a common strategy used by dominant individuals for self-benefit, and that dominant individuals are overrepresented at the top. This could naturally shift the ethical practices in the upper echelons observed in society.

**Table 5.** Measures of Dishonesty Across Studies—Dominance, Prestige, Power.

		Study 1	Study 2	Study 3	Study 4	Study 5
	Measure of dishonesty	Die throw	Virtual preprogrammed die throw	Moral disengagement	Puzzle performance	Covid-19 rule-breaking
Dominance	Pearson correlation	.255**	.222**	.216*	.180*	.138**
	Sig.	.001	.003	.010	.016	.001
	*N*	204	179	141	178	664
Prestige	Pearson correlation	.133	.039	−.123	.094	.029
	Sig.	.058	.602	.146	.214	.448
	*N*	204	179	141	178	664
Power	Pearson correlation	−	.173*	−.149	.080	.092*
	Sig.	−	.020	.078	.286	.039
	*N*	−	179	141	178	500

* Correlation is significant at the 0.05 level (2-tailed)

** Correlation is significant at the 0.01 level (2-tailed)

In contrast, tangible power was not consistently associated with dishonesty. Importantly, in naturalistic studies (Studies 2 and 5), although those with occupational power were more dishonest, this effect became insignificant when dominance was controlled for. When power was situationally induced in a high-status setting, power actually improved moral engagement (Study 3). However, when power was randomly allocated with a recall of past experiences that varied across participants, power did not impact dishonest behavior (Study 4). The findings related to power are consistent with the situated focus theory of power (Guinote, 2007a, 2008), which argues that power affects individuals in a situated manner, depending on their active contextual goals.

Like dominance, higher levels of felt prestige coincided with positions of power (Studies 2 and 5) and enjoyment of power positions (Study 3). However, unlike dominance, prestige did not predict dishonesty. These findings parallel prior research showing that prestige is associated with complaisant strategies in the pursuit of power (Ketterman & Maner, 2021).

We investigated beliefs associated with dominance that could enable dishonesty. Dominance was associated with entitlement (Studies 3 and 5) and feeling invulnerable (Study 5). These inclinations were associated with a disregard for Covid-19 containment rules (Study 5), and they seem to play a role in rule-breaking behavior (Table S5). For instance, they may justify and encourage risky behavior and engender threats to others. Finally, dominant individuals were performance oriented, which was itself related to dishonesty (Study 4). However, further research needs to investigate in more detail the proximal cognitive and emotional mechanisms that support dishonesty among dominant individuals.

### Dominance, Power, and Self-Serving Motivations

The association between dominance and dishonesty contributes to the understanding of dominance and the DBS (Johnson et al., 2012). Conceptions of dominance tend to focus mainly on power motivation (Mast et al., 2010) and the acquisition of power (Cheng et al., 2013; Maner & Case, 2016). The present findings suggest that dominance entails a desire to acquire self-benefits, with little care for norms and consequences. In particular, they raise the possibility that the drive to power observed among dominant individuals may occur because power is instrumental to acquiring freedom from constraints and monopolization of resources (Overbeck, 2010), rather than power itself being the primary reward.

As the present research shows, in the absence of competition, dominant people nevertheless strive disproportionately for personal advantages. While dominance is a relational hierarchical construct, its primary functions could be to secure resources and advantages in a social world. Indeed, dominance is linked to a heightened motivation to acquire desired outcomes, and at times referred as *resource holding power* (Zuroff et al., 2010). This conception of dominance is consistent with animal models that have defined dominance in terms of priority access to resources (e.g., food, space, and mates; Kaufmann, 1983). This provides a differentiated perspective in psychological research, which has predominantly focused on the cognitions and social strategies of dominant people in their search for power.

Dominance starts to emerge in preschool years before later stages of elaborated social or moral cognition (Guinote et al., 2015). Like in other species, early human dominance is self-serving (Boehm & Flack, 2010). As such, dominance should not necessitate abstract conceptions, such as societal ideals, to operate. However, it is possible that in adulthood dominant individuals also endorse ideologies that validate social inequalities.

Trait dominance has some overlap with social dominance orientation (SDO). SDO initially emerged within intergroup relations, independent of interpersonal dominance, as the degree to which one seeks to maintain and endorses social hierarchy or inequality (Pratto et al., 1994). Individuals high on SDO are more likely to desire occupational status (Pratto et al., 1997) and view intergroup relations as zero-sum (Sidanius et al., 1994). They tend to emerge as leaders in dyadic tasks and make unethical choices when paired with an agreeable follower (Hing et al., 2007). Leaders high on SDO can be aggressive and domineering (Lippa & Arad, 1999), and they exercise harsher influence tactics (Aiello et al., 2013). Moreover, SDO is associated with decreased awareness of corruption (Tan et al., 2016), as corruption reinforces existing social hierarchies. These connections between dominance and SDO deserve future examination.

### Power and Dishonesty

Past research failed to elucidate why instances of corruption among power holders appear common. This, we argue, is related to concentrated dominance at the top. The association between power and unethical conduct should be particularly pronounced when power is afforded by self-selections processes, whether through explicit behavior, or promotion by competition, and less so in rotation systems. The present findings are relevant in the context of employee selection and appointment of authority positions, and they contribute to research profiling those who obtain power (social class, Belmi & Laurin, 2016). Distinguishing how power is granted could be a key factor in predicting dishonesty among the powerful.

Power often magnifies the expression of personal inclinations (Guinote & Chen, 2017; Guinote et al., 2012). Here, we did not find evidence for an interaction between power and dominance on dishonesty, with the exception of a trend in Study 5.^5^ There is an important distinction between previously examined predispositions and dominance. Previously examined moral inclinations (DeCelles et al., 2012), exchange-communal orientation (Chen et al., 2001), or responsibility differences (Sassenberg et al., 2014) are not intertwined with power affordance. As dominant individuals typically have interpersonal power, this may dampen the typical effects of having situational power.

### Limitations and Directions for Future Research

While the present research shows that power does not consistently trigger dishonesty, how power affects unethical behavior deserves further scrutiny. Power is not a uniform concept. Certain types of power could increase dishonesty, whereas others may decrease dishonesty. In Study 3, when power was presented in a high-status role with responsibilities (Sassenberg et al., 2014), moral disengagement decreased. In a similar vein, the formality of power and possible differential influences on unethical behavior raise questions. Focusing on specific contexts, as demonstrated in Study 5, could provide valuable information. Power structures often enable power holders to exploit (de Cremer & van Dijk, 2005), which can affect the severity of transgressions. Even if power does not trigger more frequent dishonesty, dishonesty among the powerful may be more severe, and socially consequential.

In the present research, incentives for dishonesty and the general stakes were low. Study 4 linearly incentivized dishonesty (Fischbacher & Föllmi-Heusi, 2013) so that participants’ level of dishonesty was directly accrued self-benefit. However, it remains unclear whether the amount of incentive differentially motivates high and low power individuals. Future research could examine the effects of power when stakes are high. In addition, longitudinal studies could allow for quasi-causal claims. Selfish motivations and dishonesty are distinct concepts (Dubois et al., 2015). While the present research focused on dishonesty for self-benefit, whether the dominant would only engage in selfish (and not selfless, prosocial) forms of dishonesty warrants validation.

We focused on dominance as assertive, fearless behavior. This covers only one facet of dominance; for instance, dominance can be associated with activism and collective endeavors (Jackson, 1979). According to Wiggins’ (1979) circumplex model, dominance-submissiveness and warmth-hostility are two orthogonal axes. Keeping the dominance measurement consistent (Cheng et al., 2010) enabled us to make comparisons across studies. However, further research should broaden the scope to more positive forms of dominance that encompass leadership, warmth, and achievement motivation.

## Conclusion

We found consistent evidence that dominance is associated with elevated dishonesty, and that dominant individuals want and attain power. In contrast, power did not reliably affect dishonesty. Felt prestige, another well-defined path to power, did not predict dishonesty. If the common belief that power corrupts is ecologically valid, these findings suggest this occurs due to the overrepresentation of dominant individuals at the top. Self-selection processes rather than power per se may inherently increase the potential for dishonesty for personal gains in the high echelons.

## Supplemental Material

sj-docx-1-psp-10.1177_01461672211051481 – Supplemental material for Cheating at the Top: Trait Dominance Explains Dishonesty More Consistently Than Social Power Click here for additional data file. Supplemental material, sj-docx-1-psp-10.1177_01461672211051481 for Cheating at the Top: Trait Dominance Explains Dishonesty More Consistently Than Social Power by Kyoo-Hwa Kim and Ana Guinote in Personality and Social Psychology Bulletin

sj-docx-2-psp-10.1177_01461672211051481 – Supplemental material for Cheating at the Top: Trait Dominance Explains Dishonesty More Consistently Than Social Power Click here for additional data file. Supplemental material, sj-docx-2-psp-10.1177_01461672211051481 for Cheating at the Top: Trait Dominance Explains Dishonesty More Consistently Than Social Power by Kyoo-Hwa Kim and Ana Guinote in Personality and Social Psychology Bulletin

## Notes

1 The prestige subscale has nine items, but one was mistakenly omitted. This error was fixed in subsequent studies. Standardized values of dominance and prestige were used.

2 An examination of whether entitlement facilitates dishonest behavior among dominant individuals is discussed in Supplemental Materials (Table S3).

3 Performance motivation did *not* mediate the relationship between dominance and dishonesty (effect = .315, 95% confidence interval [CI] = [−0.0011, 0.0750], PROCESS model 4; 5,000 resamples) (Hayes, 2012).

4 Double mediation analysis with entitlement and vulnerability as mediators was significant (Figure S1). As alternative reverse models were also significant, mediation is inconclusive (see Supplemental Materials and Table S5).

5 A mini meta-analysis across Studies 2, 3, 4, and 5 showed no effects of power × dominance on dishonesty (Supplemental Materials).
