# Supplementary material for: Cheating at the Top: Trait Dominance Explains Dishonesty More Consistently Than Social Power
Source: Pers Soc Psychol Bull. 2021 Oct 16;48(12):1651–66. doi: 10.1177/01461672211051481 (PMC9596955; doi:10.1177/01461672211051481)
Supplement: sj-docx-1-psp-10.1177_01461672211051481 – Supplemental material for Cheating at the Top: Trait Dominance Explains Dishonesty More Consistently Than Social Power [file sj-docx-1-psp-10.1177_01461672211051481.docx]

**Supplemental Materials**

**Cheating at the Top: Trait Dominance Explains Dishonesty More Consistently than Social Power**

Kyoo Hwa Kim^1^, Ana Guinote^1,2^

^1^Experimental Psychology, University College London

^2^Instituto Universitário de Lisboa (ISCTE-IUL), CIS-IUL, Lisboa, Portugal

*The following information represents additional data analyses and discussions that were not included in the main document. Inserted at the end are five supplemental tables and one figure.*

**Study 1**

**Self-enhancement (mood and judgement of one’s smartness)**

*We assessed affect and self-enhancement in Studies 1 and 2.* *In order to report all data collected, we discuss the analyses on mood and smartness.*

We also hypothesized that higher dominance would be positively related to positive mood and self-enhancement, that is, feeling smart. As additional control variables, participants’ mood (happy/sad, content/discontent, relaxed/tense, good/bad) (Weick & Guinote, 2008) was recorded. Participants rated how smart they believed they were (0: *not smart at all*, 10: *smarter than anyone else*).

Dominance positively correlated with feelings of smartness *r*(204) = .357, *p* < .001, but not mood *r*(204) = -.048, *p* < .494. A multiple linear regression analysis was carried out with dominance, prestige, their interaction, as well as perceived smartness, mood, age, and gender as predictors of dishonesty. The overall regression was statistically significant *F*(7,196) = 2.423, *p* < .021, *R*^2^ = .080, Cohen’s *f*^2^ = .087. Only dominance was a significant predictor of dishonesty (dominance: *B* =.548, *p* < .006, prestige: *B* =.225, *p* < .273, dominance × prestige: *B* = -.011, *p* < .943, *smart: B = -.025, p < .852, mood: B = -.002, p < .647*, gender: *B* =.253, *p* < .222, age: *B* = -.010, *p* < .910).

**Study 2**

**Self-enhancement (mood and judgement of one’s smartness)**

*We continued to control for mood as power has been linked to positive mood (Guinote, 2017; Keltner et al., 2003) in Study 2. In order to report all data collected, we discuss the analyses on mood and smartness.*

The same measure of participants’ mood and feelings of smartness used in Study 1 were used. Holding power was not related mood *t*(177) = 1.365, *p <* .174 or perceived intelligence *t*(177) = .410, *p <* .682. Dominance was positively correlated with feelings of smartness *r*(179) = .232, *p* < .002 but not mood *r*(179) = - .065, *p* < .390.

We added control variables mood and perceived smartness to Step 1 of the stepwise multiple binary logistic regression carried out in the paper. Steps 2 and 3 were unchanged. Power significantly predicted dishonesty *B* = .366, *Wald* = 4.852, *p* < .028 although the overall regression was not significant χ^2^(5) = 7.125, *p* < .212. Adding dominance and prestige (step 2), yielded an overall significant regression χ^2^(7) = 15.178, *p* < .034. Dominance predicted dishonesty *B* = .511, *Wald* = 7.518, *p* < .006, and power was marginally related to dishonesty *B* = .308, *Wald* = 3.165, *p* < .075, while prestige was not *B* = -.081, *Wald* =.182, *p* < .670. In step 3, the model fit was worse than that of step 2, χ^2^(11) = 16.708, *p* < .117, and not significant. The results do not materially differ from the regression without mood and perceived smartness.

Dominant individuals were more likely to view themselves as smart (Studies 1 and 2). Nevertheless, this did not correlate with dishonesty.

**Study 3**

**Mediation Analysis**

In Study 3, we investigated whether dominant individuals experience greater entitlement, which in turn would facilitate moral disengagement. Feeling entitled could precede and mediate the effects of dominance on dishonesty.

***Entitlement***

Feelings of entitlement were assessed with the psychological entitlement scale (PES). The scale contained eight items, such as ‘I demand the best because I am worth it’, on 7-point Likert scales (Campbell et al., 2004; Raskin & Terry, 1988) (α = .86).

***Results***

Dominance was positively correlated with entitlement *r*(141) = .444, *p* = .001. However, entitlement was not related to moral disengagement *r*(141) = .144, *p*  = .089. We cannot conclude that entitlement explains the relationship between dominance and moral inclinations (Table S3).

**Study 5**

**Exploratory Mediation Analysis**

***Entitlement***

Dominance was positively associated to higher past *r*(664) = .104, *p* = .007, and planned *r*(664) = .133, *p* < .001, rule-breaking. It also coincided with feeling entitled *r*(664) = .307, *p* < .001, which in turn was associated with rule-breaking - both past *r*(664) = .161, *p* = .003 and planned *r*(664) = .150, *p* < .001. Past and planned rule-breaking were positively correlated *r*(664) = .494, *p* < .001, and collapsed into one variable. A bootstrapping mediation analysis was performed using PROCESS (model 4; 5000 resamples) (Hayes, 2012). Entitlement was a statistical mediator of the relationship between dominance and rule-breaking (Effect = .0254, *SE* = .0085, 95% CI [.0099, .0437]). However, reverse models were also significant. Alternative model with IV = entitlement M = dominance was significant (Effect = .0152, *SE* = .0067, 95% CI [.0027, .0290])

***Perceived vulnerability***

The higher participants’ dominance the less vulnerable to Covid-19 they felt *r*(664) = -.138, *p* < .001. Feeling vulnerable was associated with less rule-breaking, both past *r*(664) = -.340, *p* < .001 and planned *r*(664) = -.424, *p* < .001. Perceived vulnerability statistically mediated the association between dominance and rule-breaking behavior (PROCESS; Effect = .0326, *SE* = .011, 95% CI [.0123, .0539]). Alternative model with IV = vulnerability M = dominance was significant (Effect = -.0058, *SE* = .0032, 95% CI [-.0132, -.0006]). As such, the effects may be mutual, and the relationships remains tentative.

These models suggest that dominant individuals are more likely to offend because they feel entitled, and invulnerable to Covid-19 (Figure S1). Occupational power was not related to feeling entitled ŋ = -.042, nor perceived invulnerability ŋ = -.068.

**Mini Meta-Analysis**

A meta-analysis was employed to establish the robustness of the findings concerning the effects of the interaction power × dominance on dishonesty across Studies 2, 3, 4 and 5. Simple Pearson correlation coefficients weighted by sample size were employed. As the sample population was not homogeneous, Hedges-Vevea random effects model (Field & Gillett, 2010; Hedges & Vevea, 1998) was used. This analysis yielded a mean *r* of .082 and CI­_95%_ [-.008, .171], *p* = .074. The inclusion of 0 in the confidence bounds show the interactive effects of power × dominance on dishonesty are insignificant.

**Supplemental Tables and Figures**

**Table S1**

| ***Participants by Industry – Study 2***   \|  \|  \| \| Number \| \| \| --- \| --- \| --- \| --- \| --- \| \| Accommodation or food services \| \| 10 \| \| 5.6% \| \| \| Admin, support, waste management or remediation services \| \| 11 \| \| 6.1% \| \| \| Arts, entertainment or recreation \| \| 8 \| \| 4.5% \| \| \| Construction \| \| 9 \| \| 5.0% \| \| \| Educational services \| \| 27 \| \| 15.1% \| \| \| Finance or insurance \| \| 9 \| \| 5.0% \| \| \| Health care or social assistance \| \| 13 \| \| 7.3% \| \| \| Information \| \| 4 \| \| 2.2% \| \| \| Manufacturing \| \| 13 \| \| 7.3% \| \| \| Other services (except public administration) \| \| 25 \| \| 14.0% \| \| \| Professional, scientific or technical services \| \| 23 \| \| 12.8% \| \| \| Real estate or rental and leasing \| \| 4 \| \| 2.2% \| \| \| Retail trade \| \| 17 \| \| 9.5% \| \| \| Transportation or warehousing \| \| 3 \| \| 1.7% \| \| \| Unclassified establishments \| \| 1 \| \| .6% \| \| \| Wholesale trade \| \| 2 \| \| 1.1% \| \| \| Total \| \| 179 \| \| 100.0% \| \| |
| --- | --- | --- | --- | --- | --- | --- | --- | --- | --- | --- | --- | --- | --- | --- | --- | --- | --- | --- | --- | --- | --- | --- | --- | --- | --- | --- | --- | --- | --- | --- | --- | --- | --- | --- | --- | --- | --- | --- | --- | --- | --- | --- | --- | --- | --- | --- | --- | --- | --- | --- | --- | --- | --- | --- | --- | --- | --- | --- | --- | --- | --- | --- | --- | --- | --- | --- | --- | --- | --- | --- | --- | --- | --- | --- | --- | --- | --- | --- | --- | --- | --- | --- | --- | --- | --- | --- | --- | --- | --- | --- | --- | --- | --- | --- | --- | --- | --- | --- | --- | --- | --- | --- | --- | --- | --- | --- | --- |

**Table S2**

***Role Preference by Power and Prestige – Studies 3 and 4***

| **Study 3** |  |  |  | 95% Confidence Interval | |
| --- | --- | --- | --- | --- | --- |
| Prestige Level | Power Condition | Mean | Std. Error | Lower Bound | Upper Bound |
| High | High | 5.829 | .220 | 5.393 | 6.265 |
| High | Low | 5.293 | .225 | 4.848 | 5.738 |
| Low | High | 4.960 | .222 | 4.520 | 5.400 |
| Low | Low | 5.313 | .219 | 4.880 | 5.746 |
| Individuals high in felt prestige marginally preferred high power positions compared to low power positions *t*(67) = 1.982, *p* < .052. | | | | | |
| **Study 4** |  |  |  | 95% Confidence Interval | |
| Prestige Level | Power Condition | Mean | Std. Error | Lower Bound | Upper Bound |
| High | High | 4.833 | .242 | 4.355 | 5.312 |
| High | Low | 4.889 | .229 | 4.436 | 5.342 |
| Low | High | 4.830 | .253 | 4.331 | 5.330 |
| Low | Low | 4.558 | .220 | 4.123 | 4.992 |

Mean: Role preference on 7-point Likert scales. Higher mean indicates higher preference for the power condition

**Table S3**

***Associations of Moral Disengagement and Dominance, Prestige and Entitlement – Study 3***

|  | | | | Dominance | Prestige | Entitlement |
| --- | --- | --- | --- | --- | --- | --- |
| Prestige | Pearson Correlation | | .194^*^ | |  |  |
|  | Sig. (2-tailed) | | .021 | |  |  |
|  | N | | 141 | |  |  |
| Entitlement | Pearson Correlation | | .444^**^ | | .483^**^ |  |
|  | Sig. (2-tailed) | | .000 | | .000 |  |
|  | N | | 141 | | 141 |  |
| Moral Disengagement | Pearson Correlation | | .216^*^ | | -.123 | .144 |
|  | Sig. (2-tailed) | | .010 | | .146 | .089 |
|  | N | 141 | | | 141 | 141 |

*. Coefficient is significant at the 0.05 level (2-tailed).

**. Coefficient is significant at the 0.01 level (2-tailed).

**Table S4**

***Stepwise Regression on Dishonesty – Study 4***

| Model | R | R^2^ | Adjusted  R^2^ | Std. Error  of the Estimate | Change Statistics | | | | |
| --- | --- | --- | --- | --- | --- | --- | --- | --- | --- |
|  |  |  |  |  | ΔR^2^ | ΔF | df1 | df2 | Sig. ΔF |
| 1 | .131 | .017 | .000 | 1.05326 | .017 | 1.008 | 3 | 174 | .391 |
| 2 | .223 | .050 | .022 | 1.04163 | .033 | 2.953 | 2 | 172 | .055 |
| 3 | .285 | .081 | .044 | 1.03016 | .032 | 2.925 | 3 | 170 | .056 |
| 4 | .295 | .087 | .027 | 1.03913 | .006 | .270 | 4 | 166 | .897 |
| 1. Predictors: (Constant), Age, Power, Gender | | | | | | | | | |
| 2. Predictors: (Constant), Age, Power, Gender, Dominance, Prestige | | | | | | | | | |
| 3. Predictors: (Constant), Age, Power, Gender, Dominance, Prestige, Role Enjoyment, Performance Motivation | | | | | | | | | |
| 4. Predictors: (Constant), Age, Power, Gender, Dominance, Prestige, Role Enjoyment, Performance Motivation, Power × Dominance, Power × Prestige, Dominance × Prestige, Power × Dominance × Prestige | | | | | | | | | |

**Table S5**

***Associations of Rule-breaking and Power, Dominance, Prestige, Entitlement, and Perceived Vulnerability – Study 5***

|  | | | Power | Dominance | Prestige | Entitlement | Vulnerability |
| --- | --- | --- | --- | --- | --- | --- | --- |
| Rule-breaking | Pearson Correlation | .092^*^ | | .138^**^ | .029 | .180^**^ | -.444^**^ |
|  | Sig. (2-tailed) | .039 | | .000 | .448 | .000 | .000 |
|  | N | 500 | | 664 | 664 | 664 | 664 |
| Power | Pearson Correlation |  | | .189^**^ | .131^**^ | -.042 | -.068 |
|  | Sig. (2-tailed) |  | | .000 | .003 | .350 | .129 |
|  | N |  | | 500 | 500 | 500 | 500 |
| Dominance | Pearson Correlation |  | |  | .168^**^ | .307^**^ | -.138^**^ |
|  | Sig. (2-tailed) |  | |  | .000 | .000 | .000 |
|  | N |  | |  | 664 | 664 | 664 |
| Prestige | Pearson Correlation |  | |  |  | .218^**^ | -.031 |
|  | Sig. (2-tailed) |  | |  |  | .000 | .418 |
|  | N |  | |  |  | 664 | 664 |
| Entitlement | Pearson Correlation |  | |  |  |  | -.121^**^ |
|  | Sig. (2-tailed) |  | |  |  |  | .002 |
|  | N |  | |  |  |  | 664 |

*. Coefficient is significant at the 0.05 level (2-tailed).

**. Coefficient is significant at the 0.01 level (2-tailed).

**Figure S1**

***Relationship between Dominance, Entitlement, Vulnerability and Rule-breaking – Study 5*** *Model 6,* (Hayes, 2012)

-.09*

Perceived Vulnerability

Entitlement

-.11**

.06**

-.23**

.31**

| .02  .07**  Dominance  Rule-Breaking  *. Coefficient is significant at the 0.05 level (2-tailed). |
| --- |
| **. Coefficient is significant at the 0.01 level (2-tailed). |
