# Supplementary material for: Cheating at the Top: Trait Dominance Explains Dishonesty More Consistently Than Social Power
Source: Pers Soc Psychol Bull. 2021 Oct 16;48(12):1651–66. doi: 10.1177/01461672211051481 (PMC9596955; doi:10.1177/01461672211051481)
Supplement: sj-docx-2-psp-10.1177_01461672211051481 – Supplemental material for Cheating at the Top: Trait Dominance Explains Dishonesty More Consistently Than Social Power [file sj-docx-2-psp-10.1177_01461672211051481.docx]

**Methods**

**Cheating at the Top: Trait Dominance Explains Dishonesty More Consistently than Social Power**

Kyoo Hwa Kim^1^, Ana Guinote^1,2^

^1^Experimental Psychology, University College London

^2^Instituto Universitário de Lisboa (ISCTE-IUL), CIS-IUL, Lisboa, Portugal

*The following information represents a detailed description of procedures, verbatim instructions and measures used in our studies.*

**Study 1**

**Dishonesty**

You will see a dice on your desk.

Throw the dice twice, then add up the two numbers. That is the number of raffles tickets assigned to you for the lucky draw.

For example, if you throw a 5 followed by a 3, you get 8 raffles tickets.

Input the number of raffles tickets you won below.

**Participants**

52.6% (n = 87) of participants identified as Asian, and 38.2% (n = 78) of participants identified as Caucasian.

**Study 2**

**Dishonesty**

<Game of Chance>

A subset (85%) of participants will participate in an additional survey that takes 2 minutes. The participants who complete the additional survey will be paid 50p extra for their time. A quick game of chance will determine who will participate in the additional survey.

Throw the virtual die 5 times, by clicking on the button that says “Roll”.

If the sum of the 5 throws add up to 14 or more, you will be directed to the additional survey at the end of this survey.

*Statistically, 85% participants will score 14 or more when they throw a die 5 times.*

You are responsible for tracking the total of the 5 rolls to report at the end of the game.

**Occupational Power**

The triangle below represents the hierarchy in an organization. Please indicate your position in your organization, by choosing a horizontal line across the triangle at the level that best represents your position. (For example, if you position within the organization at mid-level, please choose horizontal line 4)


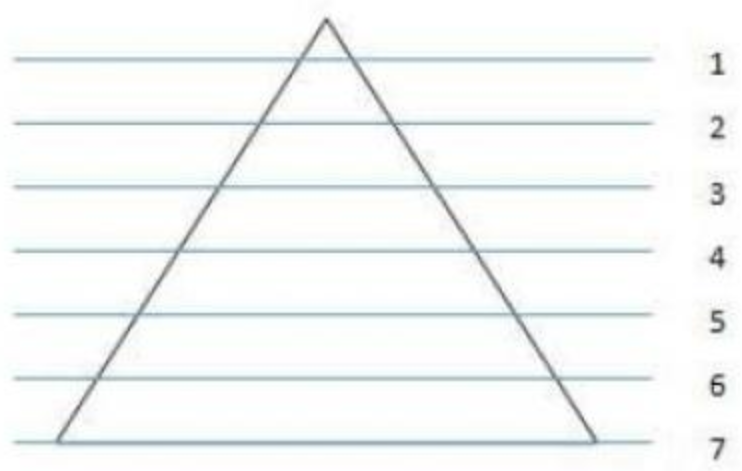


Do you hold a manager or leadership position at work? By this we mean whether you have any supervisory responsibilities such as evaluating subordinates, or having the authority to give out instructions or advice to others as part of your work.

*Yes*/*No*

*Participants who answered “Yes” were given the following additional question.*

You indicated you have supervisory responsibilities at work. How many people do you have the authority to give instructions to at work?

**Participants**

89.4% (n = 160) of participants identified as Caucasian.

**Study 3**

**Power Manipulation**

*To the managers:*

Based on your choices on the questionnaire, you have been identified as having manager skills, and thus assigned to the role of Art Gallery **Manager**. This means that you are very good at leading important projects, motivating people, and making decisions.

As the manager of the art gallery, you are responsible for the gallery's good reputation and its continued business success.

You are planning for next season's exhibition. A number of influential international critics and buyers will be visiting the gallery soon, and it is very important you set the right direction and theme for the gallery. You tell the assistant, whom you hired, what kind of work you would like him/her to do for you. You will also evaluate the assistant.

What your assistant can do for you is listed below. From this list, choose by circling 1 task you want the assistant to do for you. It is entirely up to you how you choose the task. Your assistant cannot dispute your choice, and your decision is final.

*To the assistants:*

Based on your choices on the questionnaire, you have been identified as having worker skills, and thus assigned to the role of Art Gallery **Assistant**. This means that you are good at working on tasks and following instructions from leaders.

As an assistant to the art gallery, you work for the manager, and try to satisfy the job requirement s/he has. Your boss is planning for next season's exhibition. A number of influential international critics and buyers will be visiting the gallery soon, and it is very important for your boss to set the right direction and theme for his/her gallery. Your boss (who hired you), will tell you what kind of work s/he would like you to do. Your boss will also evaluate you.

*Excerpts from the experimenter script:*

Upon completion of the study, all participants will be entered into a lottery to win vouchers. We will give out prizes to 2 participants. If you are the gallery manager and you win the lottery, your prize will be £40 in vouchers. However, it is slightly different for the assistants, and will depend on how many stars you receive. If you are an assistant, and receive 3 stars from the manager, your prize will be £60. If you get a 2-star rating, your prize will be £40. For 1-star, your prize on winning the lottery will only be £20.

**Power Motivation**

Indicate the degree to which you agree to the following statements:

I enjoyed the role that was given to me*.*

The role that was given to me suits me.

Both 7-point Likert scales (1: *strongly disagree* to 7: *strongly agree*)

**Puzzle Paradigm Instructions**

PSC (Problem Solving Capabilities) is the second project our lab in consultation with the University's curriculum team, with the objective of improving how undergraduate courses are taught. Carry out the following diagnostic test to find your level in problem-solving. You have 4 minutes to solve 6 problems. You may find some problems easier than others, but they are all solvable. If you succeed in solving 4 or more problems (out of 6), as a reward you will skip Test 2 (11 minutes) and finish EARLY!

*Puzzle task is given to students. After 4 minutes, students see the next prompt on their screen*:

Did you succeed in solving 4 or more problems? Please proceed to the next page.

(*Students not given opportunity to answer this question*)

**Moral Disengagement Questionnaire instructions**

We are investigating how people perceive and evaluate everyday behaviours. Please indicate the extent to which you agree with the following statements by ticking a circle from *(1) 'Strongly agree*' to *(7) 'Strongly disagree'* that best corresponds to your view.

There are no right or wrong answers - we are simply interested in people's perceptions.

**Participants**

51.8% (n = 73) of participants identified as Asian, and 37.6% (n = 53) of participants identified as Caucasian.

**Study 4**

**Power Manipulation**

The task you are about to complete is a writing exercise. This exercise focuses on memory, and you will be asked to recall an event from the past. Imagine you are re-living the situation provided on the next page, and write as vividly, detailed, and realistically as you can.

*The high power group:*

Please recall a particular incident in which you had power over another individual or individuals. By power, we mean a situation in which you controlled the ability of another person or persons to get something they wanted, or were in a position to evaluate those individuals. Please describe this situation in which you had power in detail - what happened, how you felt, etc.

You can take as long as you need, and write as much as you need, to complete this writing exercise. Please note you will not be able to proceed until you have written at least 600 letters, and minimum of 7 minutes.

*The low power group:*

Please recall a particular incident in which someone else had power over you. By power, we mean a situation in which someone had control over your ability to get something you wanted, or was in a position to evaluate you. Please describe this situation in which you did not have power in detail - what happened, how you felt, etc.

You can take as long as you need, and write as much you need, to complete this writing exercise. Please note you will not be able to proceed until you have written at least 600 letters, and minimum of 7 minutes.

**Dishonesty**

Those who solved two puzzles (out of six) earned 10p. The bonus increased by 10p per every puzzle, up to 50p for solving six puzzles. Participants reported their scores on a multiple-choice scale with a default pre-set at ‘6 out of 6’. They had five minutes to solve six puzzles.

<Spatial Reasoning Test>

Carry out the following diagnostic problem-solving test to find your level in problem-solving.

You will be given 4 minutes to solve 6 problems. The time limit is in place to see your ability to solve problems under time pressure.

Draw each figure **without** lifting your finger off the screen and **without retracing** any line twice:

**Performance Motivation**

I was motivated to solve more problems than other participants.

7-point Likert scale (1: *strongly disagree* to 7: *strongly agree*)

**Participants**

82% (n = 146) of participants identified as Caucasian.

**Study 5**

**Rule-breaking**

*Past rule-breaking (23 March to 15 June 2020):*

Now we ask you a few questions about the period between 23rd March and 15th June 2020, when The Government introduced measures to contain the virus in England. During this period, all shops except those providing essentials were closed.

At the end of March, Greater London area reported one of the highest rates of Covid-19 cases in the U.K.

There are no right or wrong answers. We are simply interested that you share your experience during this time.

1. During this period, on average how many times a day did you leave your home – other than for essential activities? (5-point Likert Scale, 1: *never*, 5: *more than 3 times*)
2. During this period, how many people did you meet up with in person, outside your household? (7-point Likert Scale, 1: *nobody*, 7: *more than 15*)
3. During this period, to what extent did you have physical contact (e.g. hugging, shaking hands) with someone who is not a member of your household? (7-point Likert Scale, 1: *never,* 7: *all of the time*)
4. During this period, to what extent did you adhere to the 2-meter social distancing rule? (7-point Likert Scale, 1: *all of the time,* 7: *never*)
5. During this period, how often did you wear a face covering, such as a mask outside your home? (7-point Likert Scale, 1: *all of the time,* 7: *never*)
6. During this period, how many times did you visit other households? (7-point Likert Scale, 1: *never*, 7: *more than 10 times*)

*Planned rule-breaking:*

Now we ask you a few questions about the next few weeks.

Throughout June and July 2020, The Government changed some measures in England. Greater London area no longer reported high rates of Covid-19 cases compared to the rest of the U.K.

Again, there are no right or wrong answers. We are simply interested that you share your opinions.

1. In the next 4 weeks, to what extent do you plan to adhere to the 1-meter plus social distancing rule? (7-point Likert Scale, 1: *all of the time* to 7: *never*)
2. In the next 4 weeks, how often do you plan to wear a face covering, such as a mask outside your home? (7-point Likert Scale, 1: *all of the time* to 7: *never*)
3. In the next 4 weeks, how likely is it that you will attend a gathering of more than 30 people? (7-point Likert Scale, 1: *extremely unlikely* to 7: *extremely likely*)
4. In the next 4 weeks, how likely is it that you will attend or host a gathering of more than 2 households indoors? (7-point Likert Scale, 1: *extremely unlikely* to 7: *extremely likely*)

Past and planned rule-breaking were positively correlated *r*(664) = .494, *p* < .001, and for brevity and simplicity, collapsed into one measure of Covid-19 rule-breaking. Main results of Study 5a remain consistent when rule-breaking is separated between past and planned behavior.

**Perceived Vulnerability**

7-point Likert scales (1: *strongly disagree*, 7: *strongly agree*)

1. It really bothers me when people sneeze without covering their mouths.
2. My past experiences make me believe I am NOT likely to get very sick with the Coronavirus (R).
3. I am worried about my risk of contracting Covid-19.
4. I am more likely than the people around me to catch the Coronavirus.
5. It is unlikely that I will catch the Coronavirus, even if it is going around (R).
6. It does NOT make me anxious to be around people who may have the Coronavirus (R).
7. My immune system protects me from most illnesses that other people get (R).
8. I have been afraid I would contract Covd-19.
9. I believe I already had the Coronavirus (R).

**Participants**

85.1% (n = 565) of participants identified as Caucasian.
